# Neglecting the impact of normalization in semi-synthetic RNA-seq data simulations generates artificial false positives

**DOI:** 10.1186/s13059-024-03231-9

**Published:** 2024-10-30

**Authors:** Boris P. Hejblum, Kalidou Ba, Rodolphe Thiébaut, Denis Agniel

**Affiliations:** 1 Univ. Bordeaux, INSERM Bordeaux Population Health Research Center, U1219, INRIA SISTM, Bordeaux, F-33000 France; 2 Vaccine Research Institute, Créteil, F-94000 France; 3 CHU de Bordeaux, Service d’Information Médicale, INSERM BPH, U1219, Bordeaux, F-33000 France; 4 RAND Corporation, 90401 Santa Monica, CA USA

**Keywords:** Related research article, Differential expression analysis, RNA-seq data simulation, Human population samples

## Abstract

A recent study reported exaggerated false positives by popular differential expression methods when analyzing large population samples. We reproduce the differential expression analysis simulation results and identify a caveat in the data generation process. Data not truly generated under the null hypothesis led to incorrect comparisons of benchmark methods. We provide corrected simulation results that demonstrate the good performance of dearseq and argue against the superiority of the Wilcoxon rank-sum test as suggested in the previous study.

**Supplementary Information:**

The online version contains supplementary material available at 10.1186/s13059-024-03231-9.

## Main text

Li et al. [1] recently raised significant concerns regarding popular RNA-seq differential expression methods edgeR [2] and DESeq2 [3] in the context of large human population sample sizes. We share those concerns, having ourselves come to similar conclusions [4] before, as have others [5, 6]. However, their findings that other methods (namely dearseq, limma-voom [7], and NOISeq [8]) also have increased false positive rates does not appear to be correct, and the evidence does not support their claim that the Wilcoxon rank-sum test should be preferred to these alternatives. We used the same semi-synthetic datasets that were used in Li et al. to show that no methods (including Wilcoxon test) are able to maintain the nominal level of “false discoveries” according to their definition because the data used for analysis are not truly generated under $H_0$. We demonstrate how their permutation scheme should be amended to support analysis of false positive rates under $H_0$. Using this amended scheme, we show that dearseq appears to outperform other methods under these specific settings of large human population samples and otherwise offers competitive performance on par with the other methods.

**Fig. 1:**
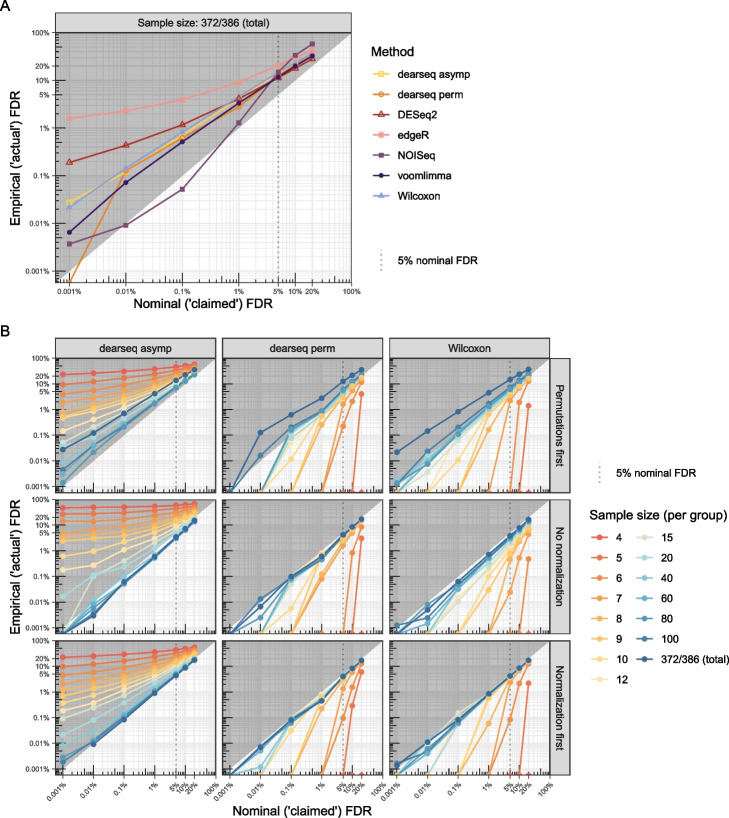
Empirical FDR control against nominal FDR level. Average over 50 semi-synthetic dataset generated from the *GTEx Heart atrial appendage VS Heart left ventricle* data. Fifty percent of the true differentially expressed (DE) genes are randomly sampled in each semi-synthetic dataset (i.e., 2889 genes remain unpermuted as true positives) and considered as gold-standard DE genes. Panel **A** reproduces the results from Li et al. [1] Fig. 2A when all methods are applied to the same data (first permuted to generate null gene expression and then normalized) on the full sample size (372 and 386 samples in each group respectively). Panel **B** studies the impact of both the sample size as well as the respective order between the data normalization and the random permutations to generate non-differentially expressed genes on the FDR control of the Wilcoxon test and on both asymptotic and permutation tests from dearseq. Of note, when applied to non-normalized data, the heteroskedasticity weights estimated by dearseq are subject to caution because observed values are then not comparable across samples

First, we demonstrate that Wilcoxon test has the same properties as the competing methods when given the same data. We recreated Fig. 2A from Li et al. where the empirical (“actual”) false discovery rate (FDR) is plotted against the nominal (“claimed”) FDR using semi-synthetic data generated from the full *GTEx Heart atrial appendage* (*n *= 372) *VS Heart left ventricle* (*n *= 386) simulation [1] in our Fig. 1A. We recomputed those results using code and data shared by the authors [9]. The key difference is that we applied the Wilcoxon test on the same normalized data (following the edgeR pipeline for filtering out genes with low counts and using log2-counts per million transformation) used by all other methods, contrary to Li et al. who conducted the Wilcoxon test on non-normalized data while conducting all other tests on normalized data. When given the same data as all other methods, the Wilcoxon test also appeared to exaggerate the FDR, as did all other methods.

This apparent increase in FDR was not due to the methods themselves, but rather to an inappropriate data-generation scheme. In Fig. 1B, we compare the performance of both dearseq asymptotic and permutation tests with the Wilcoxon test across various sample sizes (in their discussion, Li et al. [1] advocate for permutation analysis, fortunately dearseq already features such a permutation approach which we added to the comparison). In these semi-synthetic datasets, gene expression under $H_0$ was generated by randomly swapping expression values between samples. However, Li et al. did not analyze these data directly but instead normalized them before analysis. The top panel of Fig. 1B shows how the Li et al. permutation scheme leads to an apparent increase in FDR because the expression is no longer generated from $H_0$ after normalization (e.g., due to a high count being swapped into a sample with a much lower library size, artificially creating a large expression post-normalization). When the data are analyzed without normalization — an approach that would never be used in practice — we show in the middle panel of Fig. 1B that both dearseq and the Wilcoxon test attained the nominal FDR as sample size increased.

**Fig. 2:**
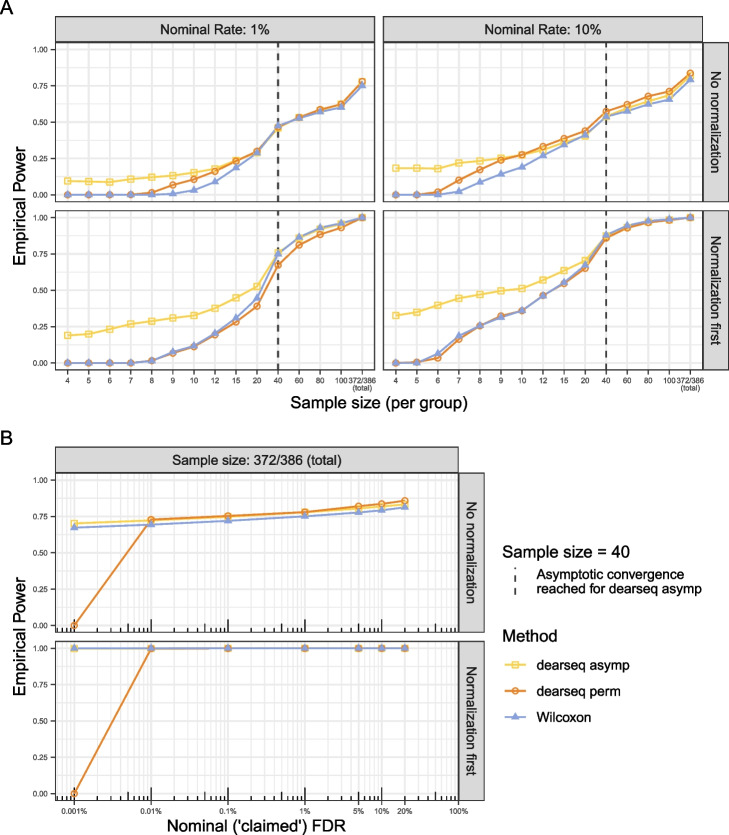
Empirical statistical power for the Wilcoxon test and dearseq asymptotic and permutation tests. Average over 50 semi-synthetic dataset generated from the *GTEx Heart atrial appendage VS Heart left ventricle* data. Fifty percent of the true differentially expressed (DE) genes are randomly sampled in each semi-synthetic dataset (i.e., 2889 genes remain unpermuted as true positives) and considered as gold standard DE genes used as true positives. Panel **A** reproduces the results from Li et al. [1] Fig. 2B as a function of sample size for both 1% and 10% nominal FDR levels, when all three methods are applied to the same data (either without any normalization or when the data are first normalized before randomly swapping values to generate expressions under $H_0$ — i.e., the two cases for which the FDR is controlled and thus the empirical power is interpretable). Panel **B** studies the impact of the nominal FDR level in both cases for the full sample size (372 and 386 samples in each group respectively)

We also show in Fig. 1B bottom panel an alternative permutation scheme which fixes the issues with the scheme in Li et al.: when counts are first normalized before being permuted under $H_0$, we demonstrate that all three tests adequately controlled the FDR for the full dataset. Figure 2 shows that once convergence was reached, the dearseq asymptotic test achieved slightly higher statistical power than Wilcoxon test, while the Wilcoxon test had superior power to dearseq permutation test (this lower power of the permutation test is largely related to the difficulty of obtaining precise estimation for the lowest *p*-values through permutations, a point of critical importance when applying a multiple-testing correction). See Additional file 1: Supplementary Fig. S3 for statistical power against empirical FDR. This amended permutation scheme should be preferred to the Li et al. permutation scheme. It is fundamental to perform differential expression analysis on samples that are normalized to ensure that expression values for a given gene are comparable across samples and in particular to remove the potential effect of library size on the analysis. The null hypothesis of interest is that there is no mean difference between conditions on the data to be analyzed, i.e., the normalized data. Thus, these are the data that should be permuted, not the raw expression, and the Li et al. permutation scheme is not informative for the desired analysis on the normalized data. Our results indicates that the apparent false positives of dearseq using the Li et al. scheme are actually detecting differences in library size. Of note, dearseq and Wilcoxon tests both display similarly good performance in Li et al.’s Fig. 1 where their permutation scheme is less problematic as all genes get permuted in that case, whereas for their Fig. 2, they introduced a confounding bias from the library size by keeping the top significant genes unpermuted (as true positive controls). See Additional file 1: Supplementary information for a detailed demonstration of the issues with library size in these data.

Both limma-voom [7] and NOISeq [8] also controlled FDR adequately using our amended permutation scheme (see Additional file 1: Supplementary Fig. S1) — note that this procedure is harder for voom-limma, edgeR [2], and DESeq2 [3] because normalization is baked into their analysis methodology. Additional file 1: Supplementary Fig. S2 shows that dearseq asymptotic test achieved higher power compared to both limma-voom and NOISeq (when $n>20$ per group).

Furthermore, dearseq is capable of handling many experimental designs beyond the simple two conditions comparison setting of the Wilcoxon test and thus constitutes a valid and versatile option for differential expression analysis of large human population samples.

## Supplementary Information

**Additional file 1:** Supplementary figures displaying results including all methods benchmarked in Li et al. [1].

## Data Availability

All code and data needed to reproduce the results presented here are openly accessible from Zenodo with DOI 10.5281/zenodo.6554347 [10].

## References

[1] Li Y, Ge X, Peng F, Li W, Li JJ. Exaggerated false positives by popular differential expression methods when analyzing human population samples. Genome Biol. 2022;23(1):79. 35292087 10.1186/s13059-022-02648-4 PMC8922736

[2] Robinson MD, McCarthy DJ, Smyth GK. edgeR: a Bioconductor package for differential expression analysis of digital gene expression data. Bioinformatics. 2010;26(1):139–40. 19910308 10.1093/bioinformatics/btp616 PMC2796818

[3] Love MI, Huber W, Anders S. Moderated estimation of fold change and dispersion for RNA-seq data with DESeq2. Genome Biol. 2014;15(12):1–21. 10.1186/s13059-014-0550-8 PMC4302049 25516281

[4] Gauthier M, Agniel D, Thiébaut R, Hejblum BP. Dearseq: a variance component score test for RNA-seq differential analysis that effectively controls the false discovery rate. NAR Genomics Bioinforma. 2020;2(4):lqaa093. 10.1093/nargab/lqaa093 PMC7676475 33575637

[5] Burden CJ, Qureshi SE, Wilson SR. Error estimates for the analysis of differential expression from RNA-seq count data. PeerJ. 2014;2:e576. 25337456 10.7717/peerj.576 PMC4179614

[6] Rocke DM, Ruan L, Zhang Y, Gossett JJ, Durbin-Johnson B, Aviran S. Excess false positive rates in methods for differential gene expression analysis using RNA-Seq data. bioRxiv. 2015. https://www.biorxiv.org/content/early/2015/06/11/020784. Accessed 4 May 2024.

[7] Law CW, Chen Y, Shi W, Smyth GK. Voom: precision weights unlock linear model analysis tools for RNA-seq read counts. Genome Biol. 2014;15(2):R29. 24485249 10.1186/gb-2014-15-2-r29 PMC4053721

[8] Tarazona S, Furió-Tarí P, Turrà D, Pietro AD, Nueda MJ, Ferrer A, et al. Data quality aware analysis of differential expression in RNA-seq with NOISeq R/Bioc package. Nucleic Acids Res. 2015;43(21):e140. 26184878 10.1093/nar/gkv711 PMC4666377

[9] Li Y, Ge X. Processed datasets for differential expression analysis on population-level RNA-seq data (Version v4) [Data set]. Zenodo. 2022. 10.5281/zenodo.6326786.

[10] Hejblum BP, Ba K, Thiébaut R, Agniel D. Datasets, reproducible codes, and results for evaluating differential expression analysis methods on population-level RNA-seq data (Version v3) [Data set]. Zenodo. 2022. 10.5281/zenodo.6554347.

