# Supplementary material for: Neglecting the impact of normalization in semi-synthetic RNA-seq data simulations generates artificial false positives
Source: Genome Biol. 2024 Oct 30;25:281. doi: 10.1186/s13059-024-03231-9 (PMC11523660; doi:10.1186/s13059-024-03231-9)
Supplement: Supplementary file 1 — Additional file 1: Supplementary figures displaying results including all methods benchmarked in Li et al. [1]. [file 13059_2024_3231_MOESM1_ESM.pdf]

Neglecting normalization impact in semi-synthetic RNA-seq data simulation generates artificial false positives
–
# Correspondence to “Exaggerated false positives by popular differential expression methods when analyzing human population samples” by Li *et al*
–
Supplementary information

Boris P Hejblum[1,2,*], Kalidou Ba[1,2], Rodolphe Thiébaut[1,2,3], Denis Agniel[4,5]

[1] *University of Bordeaux, INSERM Bordeaux Population Health Research Center, INRIA SISTM, F-33000, Bordeaux, FRANCE*
[2] *Vaccine Research Institute, F-94000, Créteil, FRANCE*
[3] *CHU de Bordeaux, F-33000, Bordeaux, FRANCE*
[4] *Department of Biomedical Informatics, Harvard Medical School, Boston, MA 02115, USA*
[5] *RAND Corporation, Santa Monica, CA 90401, USA*
[*] *to whom correspondence should be addressed: boris.hejblum@u-bordeaux.fr*

List of Figures

S1 Empirical FDR control against Nominal FDR level by method 2
S2 Empirical statistical power by method 3
S3 Empirical statistical power agianst empirical FDR by method 4
S4 Library size differences 5
S5 Library size of true DE only 6
S6 Boxplots of Wilcoxon test p-values in repeated toy examples 8

S1 Supplementary figures of performance evaluation

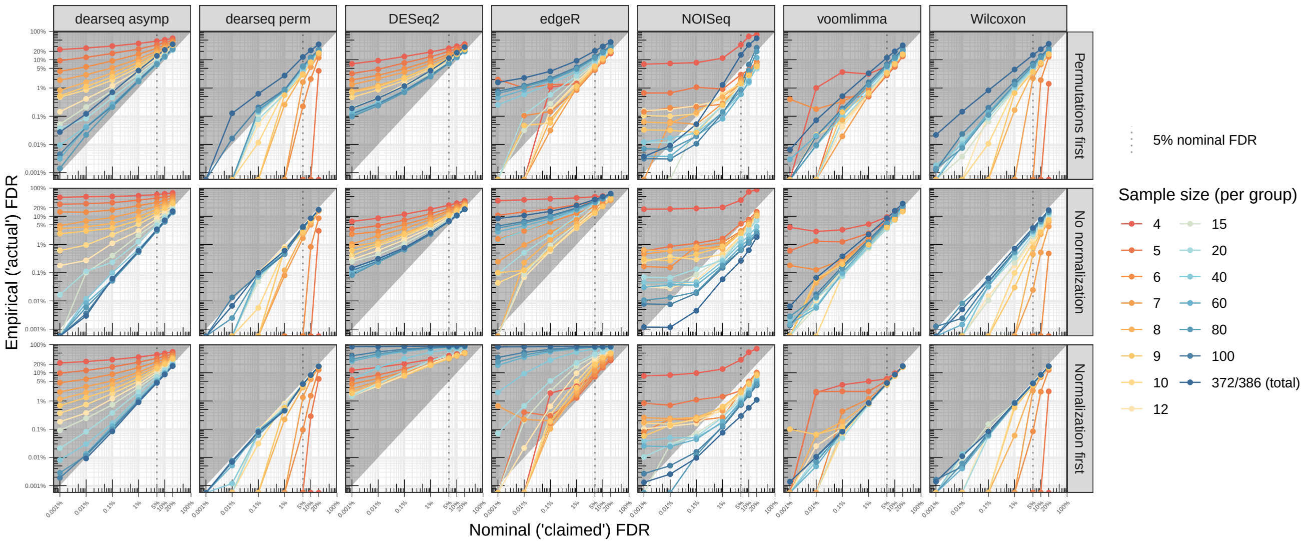

Figure S1: **Empirical FDR control against Nominal FDR level by method** Average over 50 semi-synthetic dataset generated from the *GTEx Heart atrial appendage VS Heart left ventricle* data. 50% of the true Differentially Expressed (DE) genes are randomly sampled in each semi-synthetic dataset (i.e. 2,889 genes remains unpermuted as true positives) and considered as gold-standard DE genes. **First row** reproduces the results from figure 2A in Li et al.'s[1] when all methods are applied to the same data (first permuted to generate null gene expression and then normalized, inducing false positives) at various sample sizes (in each group). **Second row** display the same results when the permuted data are analyzed without doing normalization at all for any methods. **Third row** considers the amended simulation scenario where data are first normalized and then permuted to generate expression under H_0, in which case only `DESeq2` and `edgeR` still feature exaggerated false positives.

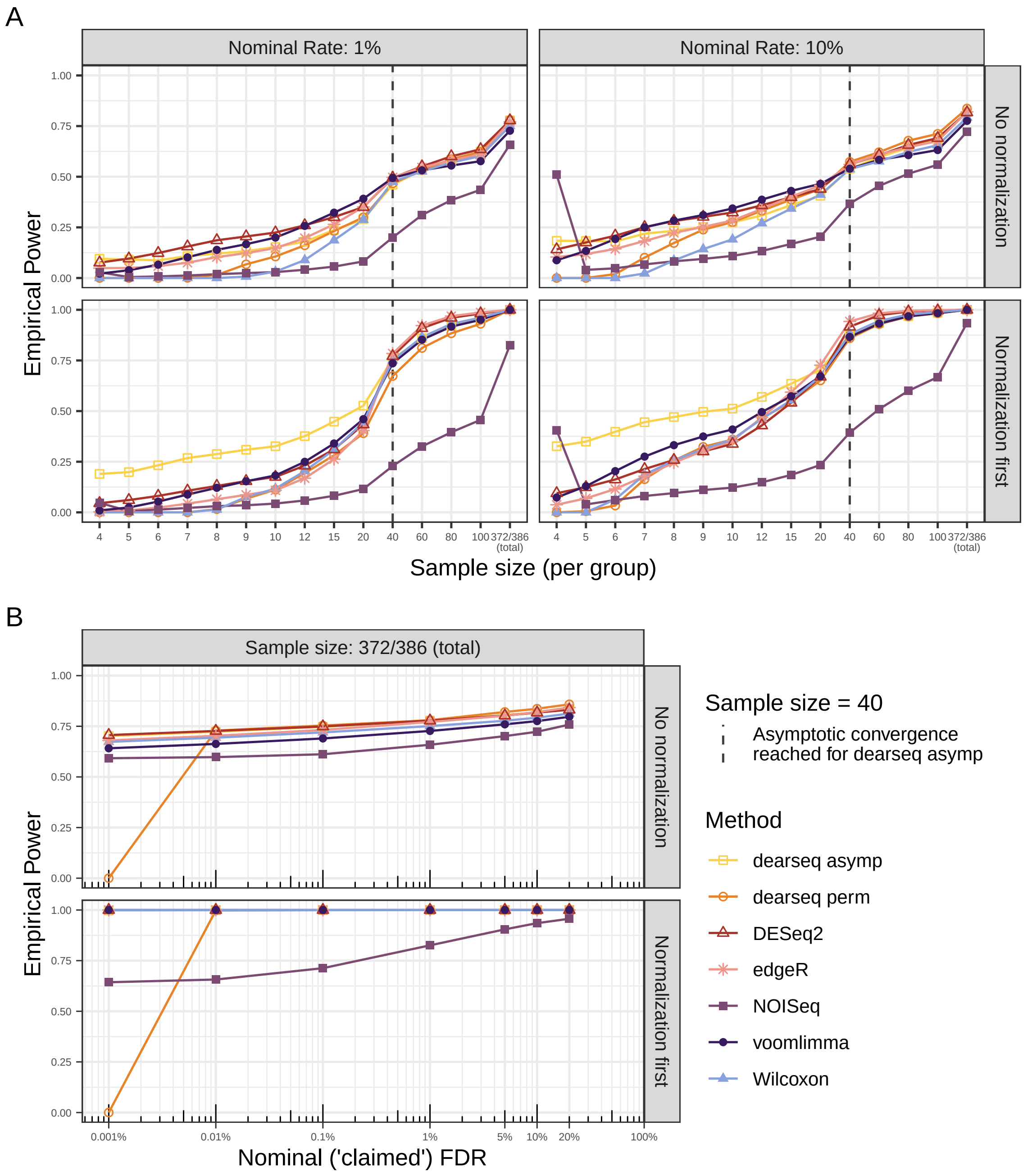

Figure S2: **Empirical statistical power by method.** Average over 50 semi-synthetic dataset generated from the *GTEx Heart atrial appendage VS Heart left ventricle* data. 50% of the true Differentially Expressed (DE) genes are randomly sampled in each semi-synthetic dataset (i.e. 2,889 genes remains unpermuted as true positives) and considered as gold-standard DE genes used as true positives. NB: for the empirical power to be interpretable, FDR control is warranted: thus **edgeR** and **DESeq2** results should not be interpreted here. **Panel A** reproduces the results from Li et al.[1] Figure 2B as a function of sample size for both 1% and 10% nominal FDR levels, when all methods are applied to the same data (either without any normalization or when the data are first normalized before randomly swapping values to generate expressions under H_0). **Panel B** study the impact of the nominal FDR level in both cases for the full sample size (372 and 386 samples in each group respectively).

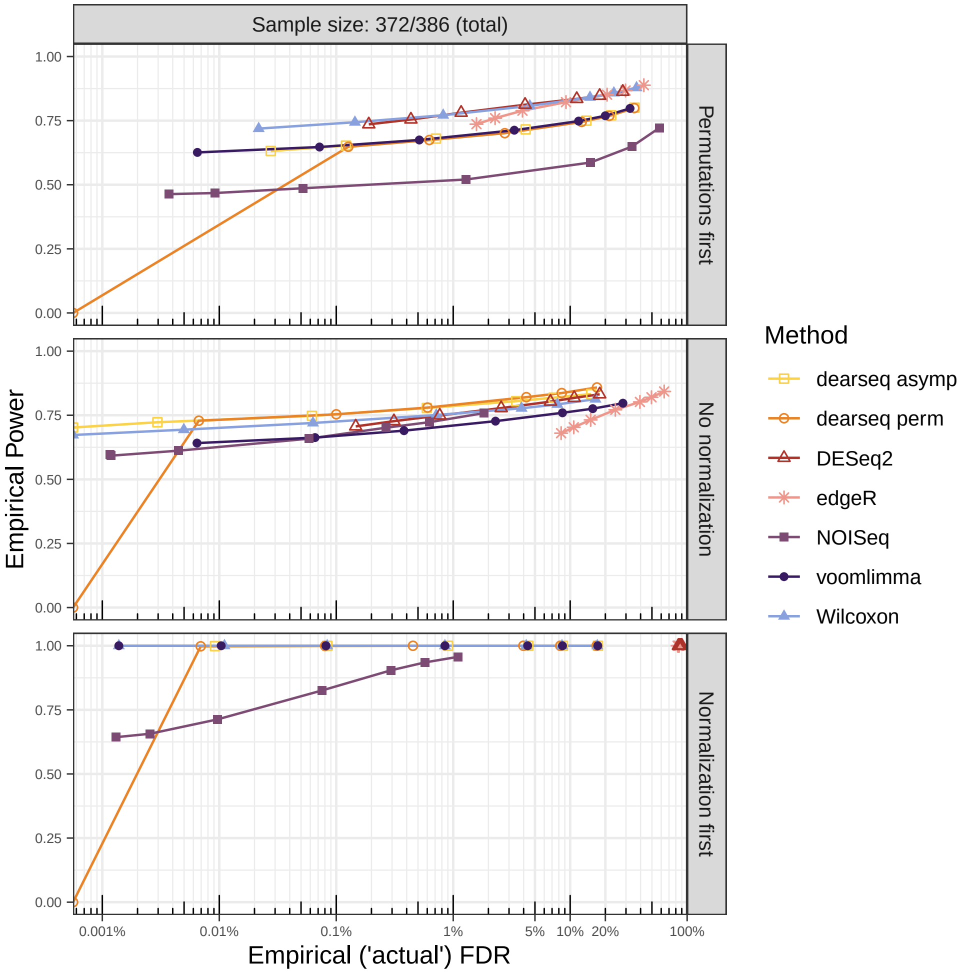

Figure S3: **Empirical statistical power agianst empirical FDR by method.** Average over 50 semi-synthetic dataset generated from the *GTEx Heart atrial appendage VS Heart left ventricle* data for the full sample size (372 and 386 samples in each group respectively). 50% of the true Differentially Expressed (DE) genes are randomly sampled in each semi-synthetic dataset (i.e. 2,889 genes remains unpermuted as true positives) and considered as gold-standard DE genes used as true positives.

Impact of library size and normalization

The main source of false positives generated in the *permutation first* scheme is likely the difference in library size. Contrary to when all genes are permuted (cf the analysis presented by Li *et al*[1] in their Figure 1 where neither `dearseq` nor `limma-voom` or `NOISeq` suffer from false positive inflation), when some genes – *a fortiori* differentially expressed (DE) genes – are left unpermuted, a difference in library size between the two conditions of interest can subsist even after the permutation. In such case, this library size difference will affect the normalization.

Figure S4 displays and characterizes the imbalance of library sizes between the two heart tissues from the *GTEx Heart atrial appendage VS Heart left ventricle* dataset used in this example. Figure S5 shows that this imbalance is mainly conserved in the subset of 5,778 genes that are considered as truly DE by Li *et al* (the intersections of genes that are significantly DE according to all five methods `DESeq2`, `edgeR`, `NOISeq`, `limma-voom` and Wilcoxon test at a FDR threshold of 10^{-6}). This also explains results from their supplementary Figure S19 where the higher the proportion of true DE genes, the more false positives are generated by this library size difference remaining after their permutation scheme.

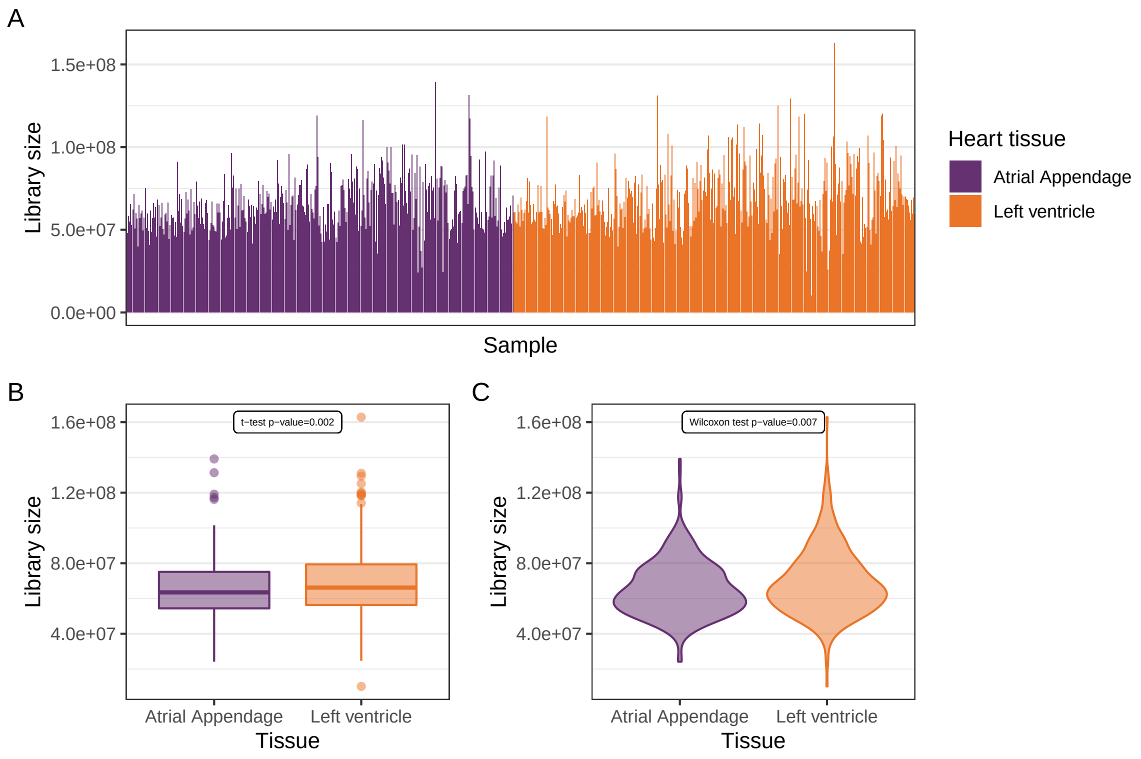

Figure S4: **Library size differences in the *GTEx Heart atrial appendage VS Heart left ventricle* data. Panel A** displays the library sizes of all 758 samples (372 and 386 in the atrial appendage and left ventricule heart tissues respectively). **Panel B** presents a boxplot highlighting the statistically significant difference with a t-test. **Panel C** presents a violin plot for a non-parametric comparison with the Wilcoxon test.

Toy example

Here we present a toy example to illustrate the impact of post-permutation normalization due to library size imbalance.

Consider three genes: *Gene 1* and *Gene 3* are truly associated with the condition of interest A, while *Gene 2* is independent of this condition. These three genes are measured across 10 samples with a library size that varies from 80 to 15,000. Table S1 presents the raw data while Tables S2, S3, and S4 display the normalized, permuted and normalized permuted data respectively. We observe that

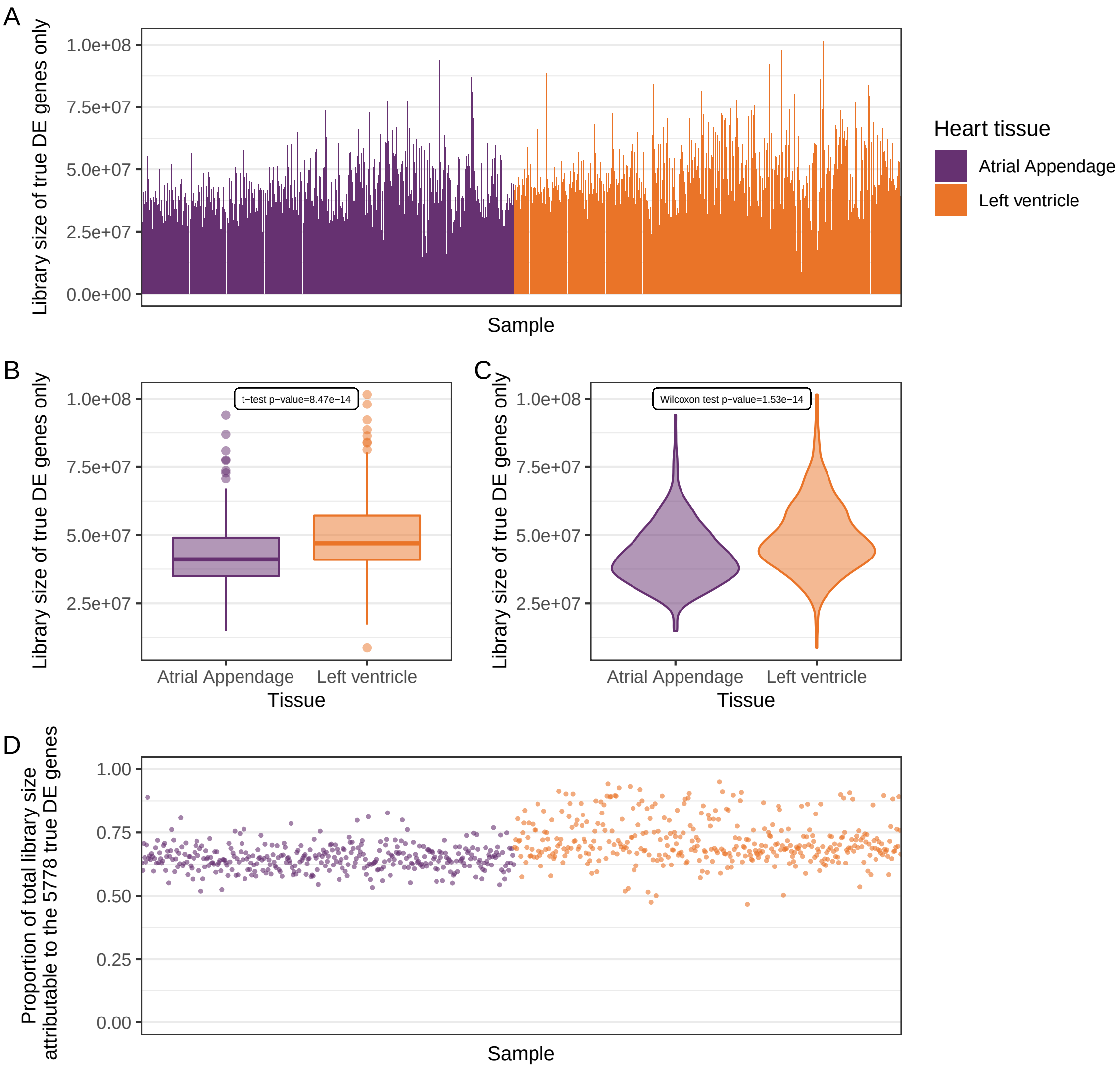

Figure S5: **Library size of true DE genes only in the *GTEx Heart atrial appendage VS Heart left ventricle* data. Panel A** displays the library sizes of all 758 samples (372 and 386 in the atrial appendage and left ventricule heart tissues respectively) when only using the 5,778 true DE genes. **Panel B** presents a boxplot highlighting the statistically significant difference with a t-test. **Panel C** presents a violin plot for a non-parametric comparison with the Wilcoxon test. **Panel D** shows that most of the total library size is accounted for by the subset of the 5,778 true DE genes, and even more so for the left ventricle heart tissue.

in this example there is confusion between the condition and the library size (i.e. not normalizing the data leads to *Gene 2* being significantly associated with condition A, while normalizing by $\widetilde{y}_{ij} = \log 2(\frac{y_{ij}}{\sum_{i=1}^{10} y_{ij}})$ removes the spurious association). Following the Li *et al*[1] permutation scheme, we only permute *Gene 2*, and we keep *Gene 1* and *Gene 3* unchanged as both are true DE genes. While the permuted *Gene 2* is not associated to condition A (as expected), the permutation has swapped values coming from various library sizes: due to library size differences, expression values are non-exchangeable across samples under the null, as can be seen in Table S3. And because of *Gene 1* and *Gene 3* remaining unpermuted, the new library sizes are quite close to the original ones, still correlated to the condition of interest. Normalizing to account for library sizes that do not match with the swapped values in *Gene 2* now has the unwanted effect of adding back differences between the two conditions due to the confusion with the library size, as shown in Table S4.

Library size	Condition	Gene 1	Gene 2	Gene 3
80	0	8	16	57
100	0	12	20	70
150	0	16	34	106
500	0	58	108	356
800	0	93	169	566
1,000	1	404	211	408
5,000	1	2,050	1,052	2,042
5,000	1	2,062	1,050	2,047
10,000	1	4,104	2,094	4,092
15,000	1	6,134	3,165	6,163

Table S1: **Original data.** Wilcoxon test p-value for Gene 2 between the two conditions is 0.008.

Library size	Condition	Gene 1	Gene 2	Gene 3
80	0	-3.32	-2.32	-0.49
100	0	-3.06	-2.32	-0.51
150	0	-3.23	-2.14	-0.50
500	0	-3.11	-2.21	-0.49
800	0	-3.10	-2.24	-0.50
1,000	1	-1.31	-2.24	-1.29
5,000	1	-1.29	-2.25	-1.29
5,000	1	-1.28	-2.25	-1.29
1,0000	1	-1.28	-2.26	-1.29
15,000	1	-1.29	-2.24	-1.28

Table S2: **Normalized data.** Wilcoxon test p-value for Gene 2 between the two conditions is 0.674.

Library size	Condition	Gene 1	Gene 2	Gene 3
234	0	8	169	57
1,134	0	12	1,052	70
3,287	0	16	3,165	106
448	0	58	34	356
870	0	93	211	566
828	1	404	16	408
4,112	1	2,050	20	2,042
5,159	1	2,062	1,050	2,047
10,290	1	4,104	2,094	4,092
12,405	1	6,134	108	6,163

Table S3: **Permuted data.** Wilcoxon test p-value for Gene 2 between the two conditions is 0.421.

Library size	Condition	Gene 1	Gene 2	Gene 3
234	0	-4.87	-0.47	-2.04
1134	0	-6.56	-0.11	-4.02
3287	0	-7.68	-0.05	-4.95
448	0	-2.95	-3.72	-0.33
870	0	-3.23	-2.04	-0.62
828	1	-1.04	-5.69	-1.02
4112	1	-1.00	-7.68	-1.01
5159	1	-1.32	-2.30	-1.33
10290	1	-1.33	-2.30	-1.33
12405	1	-1.02	-6.84	-1.01

Table S4: **Normalized permuted data.** Wilcoxon test p-value for Gene 2 between the two conditions is 0.031.

When repeating this toy example 500 times, the Wilcoxon test on normalized permuted data clearly has an inflated Type-I error as showed in Figure S6 compared to the uniform distribution expected under the null (p-values are uniform for the normalized data as well as for the permuted data, as expected).

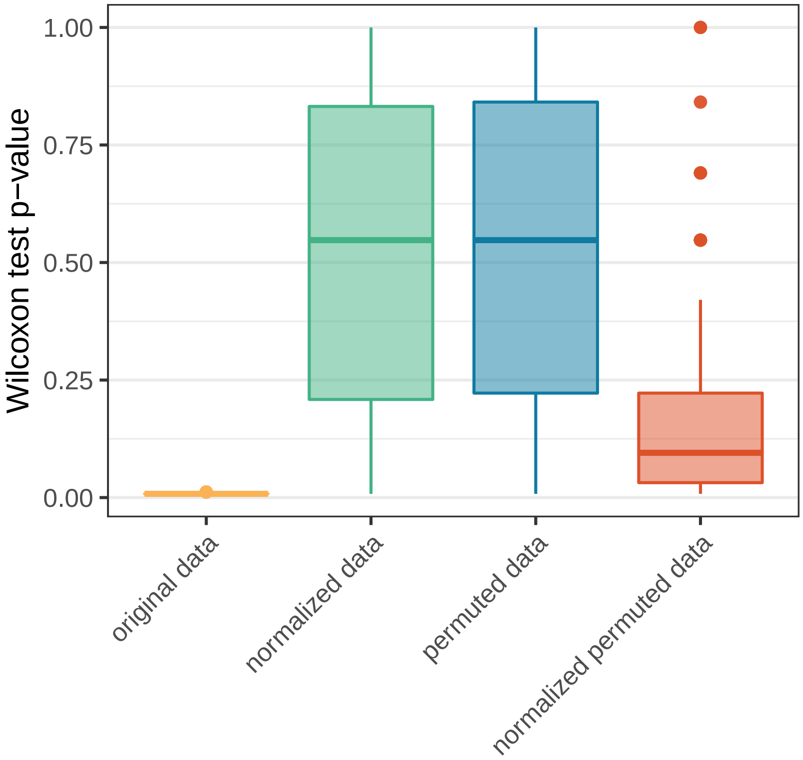

Figure S6: **Boxplots of Wilcoxon test p-values according to the data processing in 500 repetitions of the toy example.**

References

[1] Li Y, Ge X, Peng F, Li W, Li JJ. Exaggerated False Positives by Popular Differential Expression Methods When Analyzing Human Population Samples. Genome Biology. 2022;23(1):79.
